# Influence of Annealing Process on Soft Magnetic Properties of Fe-B-C-Si-P Amorphous Alloys

**DOI:** 10.3390/ma17061447

**Published:** 2024-03-21

**Authors:** Jili Jia, You Wu, Lingxiang Shi, Ranbin Wang, Wenhui Guo, Hengtong Bu, Yang Shao, Na Chen, Kefu Yao

**Affiliations:** 1 School of Materials Science and Engineering, Tsinghua University, Beijing 100084, China; 2 Department of Mechanical Engineering, City University of Hong Kong, Tat Chee Avenue, Kowloon, Hong Kong

**Keywords:** amorphous alloy, long-time annealing, soft magnetic properties, magnetic domain

## Abstract

It is well known that the annealing process plays a key role in tuning the properties of Fe-based amorphous soft magnetic alloys. However, the optimal annealing process for a particular amorphous alloy is often difficult to determine. Here, Fe_81.4_B_13.2_C_2.8_Si_1.8_P_0.8_ and Fe_82.2_B_12.4_C_2.8_Si_1.8_P_0.8_ amorphous alloys (denoted as Fe_81.4_ and Fe_82.2_) were prepared to systematically study the effects of the annealing temperature and time on the soft magnetic properties. The results show that the optimum annealing temperature ranges of the Fe_81.4_ and Fe_82.2_ amorphous alloys were 623 K to 653 K and 593 K to 623 K, and their coercivity (*H_c_*) values were only 2.0–2.5 A/m and 1.3–2.7 A/m, respectively. Furthermore, a characteristic temperature *T_ai_* was obtained to guide the choosing of the annealing temperature at which the *dB_s_*/*dT* begins to decrease rapidly. Based on the theory of spontaneous magnetization, the relationship between *T_ai_* and the optimum annealing temperature ranges was analyzed. When the annealing temperature was higher than *T_ai_*, the effect of the internal magnetic field generated by spontaneous magnetization on the relaxation behavior was significantly reduced, and the alloys exhibited excellent soft magnetic properties. It is worth indicating that when annealed at 603 K (slightly higher than *T_ai_*), the Fe_82.2_ amorphous alloys exhibited excellent and stable soft magnetic properties even if annealed for a long time. The *H_c_* of Fe_82.2_B_12.4_C_2.8_Si_1.8_P_0.8_ amorphous alloys was only 1.9 A/m when annealed at 603 K for 330 min. This value of *T_ai_* is expected to provide a suggestion for the proper annealing temperature of other amorphous soft magnetic alloys.

## 1. Introduction

Among the diverse soft magnetic materials, Fe-based amorphous alloys have attracted intensive attention due to their highly comprehensive soft magnetic performance, including low coercivity (*H_c_*) and core loss (W) [1,2,3,4]. The structure of Fe-based amorphous alloys obtained through rapid solidification has various inhomogeneities, such as loose and dense atomic packing regions, regions with different internal stresses, and magnetic heterogeneous regions [5]. The *H_c_* of Fe-based amorphous alloys is directly related to the inhomogeneity, internal stress, and free volume of amorphous alloys [5,6,7,8]. In order to reduce *H_c_* and improve the soft magnetic properties, annealing is generally carried out near or above the Curie temperature (*T_C_*) of amorphous alloys [5,9]. Above *T_C_*, amorphous alloys are paramagnetic, so the stress release and structural relaxation are no longer affected by the local internal magnetic field [5,9,10]. Therefore, the magnetic domain structure is optimized, thus greatly improving the soft magnetic properties.

At present, the annealing time of amorphous alloys reported in the literature is relatively short, especially for those with a relatively high iron content; most of them are no more than 30 min [9,11,12,13,14,15]. However, for the industrial production of amorphous alloy products, long-time annealing is of great importance. Due to the characteristics of the furnace structure and the large volume of materials, the time required for uniform temperature distribution in the industrial production process is longer than that under experimental conditions. The time required to reach the set temperature for different locations in the furnace is different, but the properties of materials annealed in the same batch need to be as similar as possible. Therefore, it is required that the properties of amorphous alloys be kept stable during the annealing time for as long as possible.

A few studies have suggested that when annealing for a long time, even if the annealing temperature is slightly lower than *T_C_*, good soft magnetic properties can also be obtained [16,17]. Thus, the question of how to determine the optimal annealing temperature for long-time annealing is important. *T_C_* is a key temperature parameter used to describe the property transition of magnetic materials from ferromagnetic to paramagnetic; it is often used as a criterion for selecting the optimal annealing temperature in short-time annealing. Similarly, it is important to find a way to determine the optimal annealing temperature in long-time annealing and to understand the variation in magnetic properties with temperature.

In this study, Fe_81.4_B_13.2_C_2.8_Si_1.8_P_0.8_ and Fe_82.2_B_12.4_C_2.8_Si_1.8_P_0.8_ amorphous alloys with relatively high Fe content were designed and prepared. The effects of the annealing temperature and time on the soft magnetic properties, magnetic domain structure, and magnetization process of the amorphous alloys were systematically studied. The correlation between the magnetic properties and the structure of the annealed amorphous alloys is discussed, and the process for the magnetic property adjustment of Fe-based amorphous alloys in long-time annealing is investigated.

## 2. Materials and Methods

Amorphous alloy ribbons with nominal compositions of Fe_81.4_B_13.2_C_2.8_Si_1.8_P_0.8_ (denoted as Fe_81.4_) and Fe_82.2_B_12.4_C_2.8_Si_1.8_P_0.8_ (denoted as Fe_82.2_) were produced with single-roller spinning. The thermal properties of the amorphous alloys were examined using differential scanning calorimetry (DSC, Netzsch STA 449 F3, Netzsch, Selb, Germany) at a heating rate of 40 K/min. The temperature dependence of magnetization of the amorphous alloys was measured using superconducting quantum interference device (SQUID, MPMS-3, Quantum Design, San Diego, CA, USA) magnetometry under an applied field of 800 kA/m at a heating rate of 10 K/min. The ribbons, with a width of about 1 mm and a thickness of about 25 μm, were cut to an 80 mm length for subsequent annealing and measurements. The structures of the as-spun and annealed samples were identified using X-ray diffraction (XRD, Rigaku D/max 2500, Rigaku, Tokyo, Japan) with Cu Kα radiation. The structure of the as-spun Fe_82.2_ amorphous alloy ribbon was also identified using high-resolution transmission electron microscopy (HRTEM, FEI Tecnai G2 F20, FEI, Hillsboro, OR, USA). The static magnetization curves and hysteresis loops were measured using a DC B-H hysteresis loop tracer (Linkjoin MATS-2010SD, Linkjoin, Loudi, China) under a field of 800 A/m. The domain structures were observed with a magneto-optical Kerr microscope (MOKE, Zeiss Imager D2m, Zeiss, Oberkochen, Germany).

In order to explore the effect of the annealing process on the soft magnetic properties of amorphous alloys, isothermal annealing treatments of the amorphous ribbons were carried out with the normal annealing (NA) process. This consisted of the following three steps: (1) the ribbon sample was fixed in a copper holder and then sealed in a quartz tube filled with argon gas; (2) the temperature inside the electric tube furnace was raised to the set value, and the quartz tube was pushed into the furnace; (3) after holding in the furnace for a given time, the quartz tube was pulled out of the furnace and quenched in water to room temperature.

## 3. Results and Discussion

**Figure 1:**
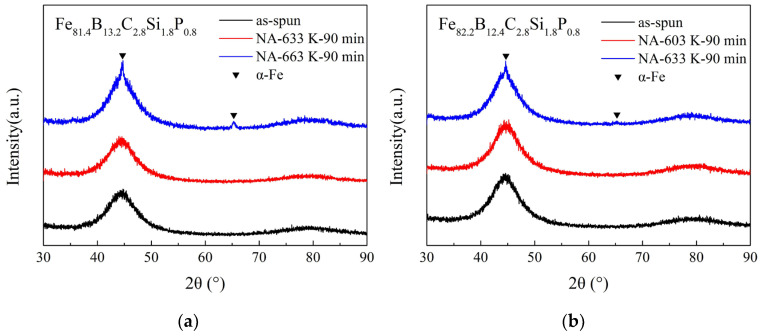
Smooth-side XRD patterns of as-spun ribbons and ribbons with different annealing temperatures for (**a**) Fe_81.4_B_13.2_C_2.8_Si_1.8_P_0.8_ and (**b**) Fe_82.2_B_12.4_C_2.8_Si_1.8_P_0.8_ amorphous alloys.

**Figure 2:**
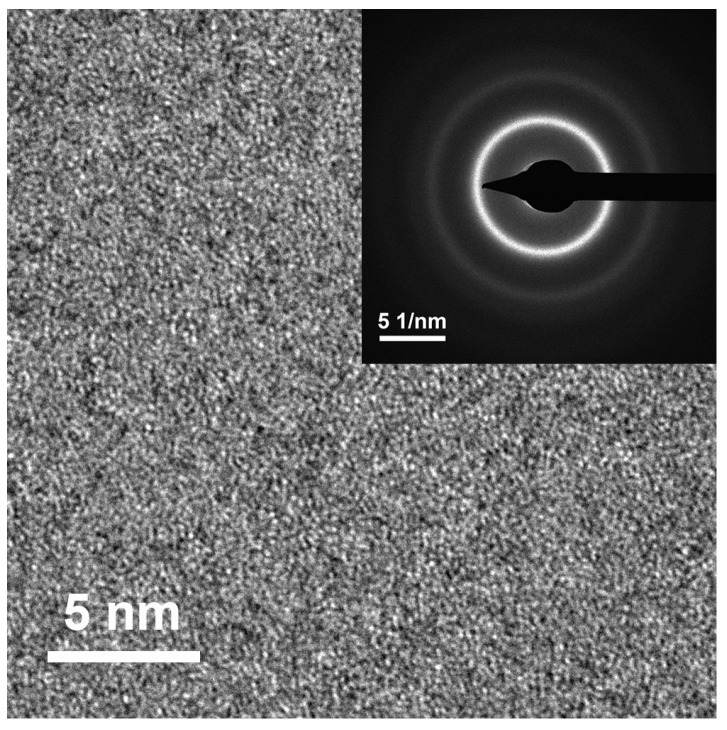
High-resolution transmission electron microscope (HRTEM) image and selected-area electron diffraction (SAED) pattern of Fe_82.2_B_12.4_C_2.8_Si_1.8_P_0.8_ amorphous alloy.

**Figure 3:**
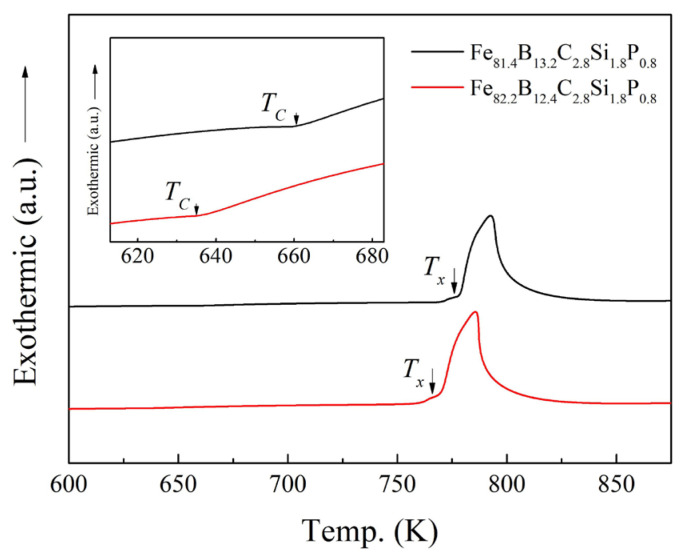
DSC curves of Fe_81.4_B_13.2_C_2.8_Si_1.8_P_0.8_ and Fe_82.2_B_12.4_C_2.8_Si_1.8_P_0.8_ amorphous alloys at a heating rate of 0.67 K/s. The inset shows an enlarged section of the curves around the Curie temperature *T_C_*.

In order to enhance the saturation magnetic flux density (*B_s_*) of Fe-based amorphous alloys, the Fe content should be increased in the alloys. Based on former research results and considering the synergetic effect of metalloid elements B, C, Si, and P [17,18,19,20,21], two alloys with nominal compositions of Fe_81.4_B_13.2_C_2.8_Si_1.8_P_0.8_ and Fe_82.2_B_12.4_C_2.8_Si_1.8_P_0.8_ were designed and prepared via single-roller spinning. The structure of the prepared alloy ribbons was examined using the XRD method. As shown in Figure 1, no sharp diffraction peak corresponding to the crystalline phases in the XRD patterns of the as-spun Fe_81.4_ and Fe_82.2_ ribbons (black lines in Figure 1a and Figure 1b, respectively) can be observed, indicating that both of them possessed an amorphous structure. The HRTEM image and SAED pattern in Figure 2 further confirm the amorphous nature of the as-spun Fe_82.2_ ribbon. This implies that the designed alloys possessed good glass-forming abilities. The thermal properties of the as-spun ribbons, including the Curie temperature (*T_C_*) and the onset temperature of crystallization (*T_x_*), were determined from the DSC curves shown in Figure 3. The *T_C_* and *T_x_* of the Fe_81.4_ amorphous ribbons were 661 K and 776 K, respectively, and those of Fe_82.2_ were 635 K and 766 K, respectively. This indicates that the value of *T_x_*−*T_C_* was over 110 K. When the Fe content was slightly increased by 0.8 at.% (the B content was reduced by 0.8 at.%), the *T_C_* and *T_x_* of the Fe-based amorphous alloy were clearly reduced. The decrease in the *T_x_* is due to the decrease in thermal stability caused by the increase in Fe content and the decrease in B content [22,23]. Meanwhile, the decrease in *T_C_* is due to the decrease in the exchange interaction [18,24].

**Figure 4:**
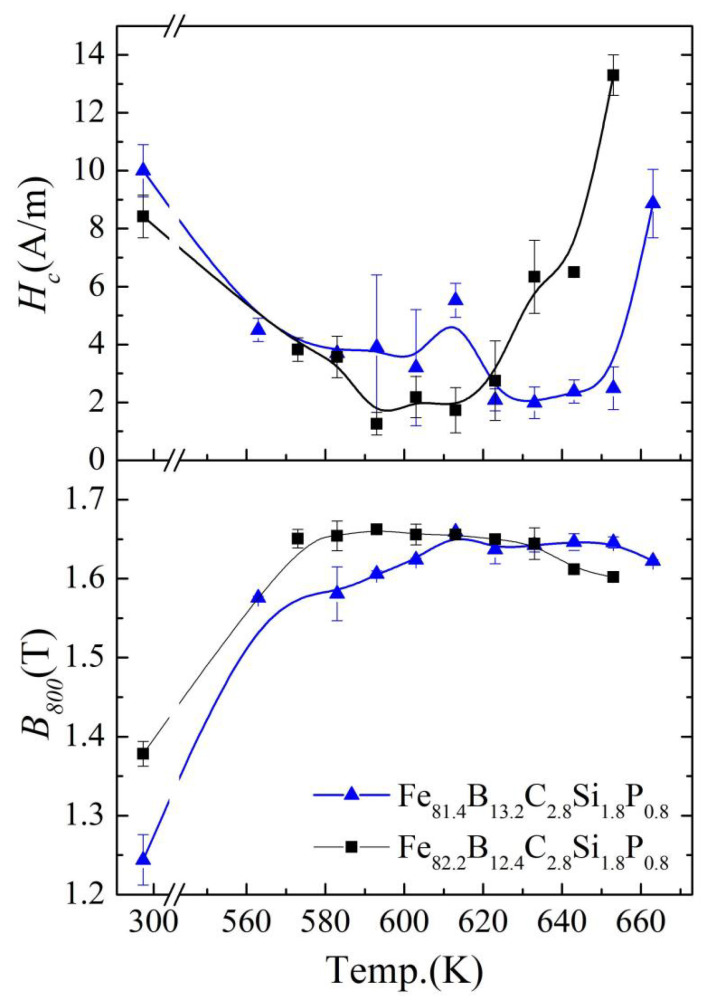
Annealing temperature dependence of *H*_c_ (coercivity) and *B*_800_ for the Fe_81.4_B_13.2_C_2.8_Si_1.8_P_0.8_ and Fe_82.2_B_12.4_C_2.8_Si_1.8_P_0.8_ amorphous alloy ribbons after normal annealing for 90 min.

The amorphous ribbons were annealed at different temperatures from 563 K to 663 K with an interval of 10 K. Figure 4 shows the annealing temperature dependence of coercivity (*H_c_*) and the magnetic flux density measured at an applied magnetic field of 800 A/m (*B*_800_) for the Fe_81.4_B_13.2_C_2.8_Si_1.8_P_0.8_ and Fe_82.2_B_12.4_C_2.8_Si_1.8_P_0.8_ amorphous alloy ribbons after normal annealing for 90 min. With the increase in the annealing temperature, the *H_c_* decreased to a low point at first, then maintained a stable state and finally increased rapidly. The stable region with the lowest *H_c_* obtained in the experiment is defined as the optimal annealing temperature range. The lowest temperature of the optimum annealing temperature range is denoted as *T_a_*_0_, and the highest temperature is denoted as *T_am_*. The *T_a_*_0_ and *T_am_* of the Fe_81.4_ amorphous alloy were 623 K and 653 K, respectively, while the *T_a_*_0_ and *T_am_* of the Fe_82.2_ amorphous alloy were 593 K and 623 K, respectively. As the annealing temperature increased, the *B*_800_ of the alloys initially increased then reached a plateau and finally declined. *B*_800_ is a comprehensive reflection of the *B_s_*, *H_c_,* and permeability [25]. The initial temperatures of the *B*_800_ plateau were 613 K and 573 K for Fe_81.4_ and Fe_82.2_, respectively, which are lower than *T_a_*_0_. However, the end temperature of the *B*_800_ plateau was equal to *T_am_*. The reduction in *H_c_* was due to the relaxation of stress and the reduction in the free volume [6]. However, spontaneous magnetization can affect the relaxation progress of amorphous alloys [5]. Therefore, the beginning of the optimum annealing temperature range of *H_c_* did not coincide with the starting temperature of relaxation. The present results show that both the Fe_81.4_ and Fe_82.2_ amorphous alloys exhibited an optimized annealing temperature range of 30 K, together with *B*_800_ and *H_c_* values of 1.64–1.65 T and 2.0–2.5 A/m and 1.65–1.66 T and 1.3–2.7 A/m, respectively. This indicates that, after annealing, the developed amorphous alloys were excellent soft magnetic materials, exhibiting a high *B*_800_ and low *H_c_* (Figure 4).

**Figure 5:**
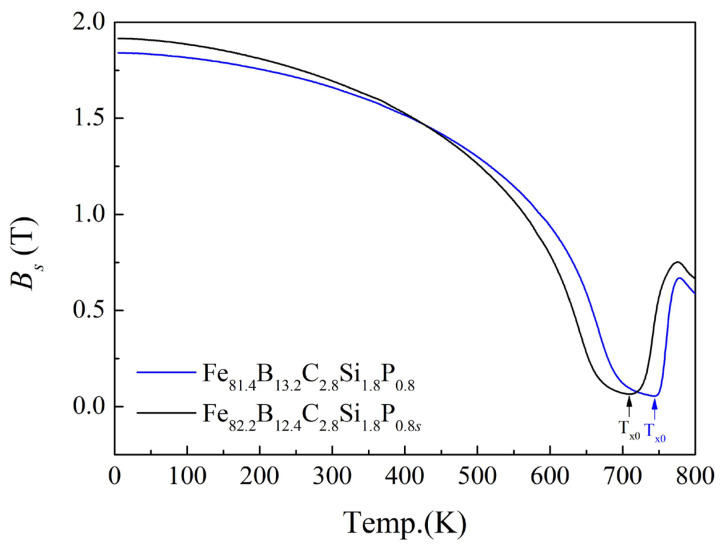
Temperature dependence of *B_s_* for Fe_81.4_B_13.2_C_2.8_Si_1.8_P_0.8_ and Fe_82.2_B_12.4_C_2.8_Si_1.8_P_0.8_ amorphous alloys.

In order to understand the effect of elements on the magnetic property, the temperature dependence of the saturation flux density (*B_s_*) for the Fe_81.4_ and Fe_82.2_ amorphous alloys was obtained and studied (Figure 5). At a temperature of 5 K, the *B_s_* values of the Fe_81.4_ and Fe_82.2_ amorphous alloys were 1.84 T and 1.92 T, respectively. At a temperature of 5 K, the *B_s_* values of the Fe_81.4_ and Fe_82.2_ amorphous alloys were 1.84 T and 1.92 T, respectively. When the Fe content increased, the proportion of ferromagnetic atoms increased; thus, the total magnetic moment increased, leading to an increase in the *B_s_* near 0 K [18,26]. As the temperature increased, the atomic thermal motion increased. In ferromagnetic materials, the thermal motion of atoms disturbs the spontaneous magnetization of atomic magnetic moments [24]. As a result, the *B_s_* of the Fe_81.4_ and Fe_82.2_ amorphous alloys decreased continuously with the increase in temperature, until it reached the lowest point. The temperature with the lowest *B_s_* is denoted as *T_x0_*. When the temperature exceeded *T_x_*_0_, the *B_s_* began to increase, which was caused by crystallization of the amorphous alloys. Finally, the *B_s_* decreased again because the spontaneous magnetization of α-Fe decreased rapidly. In a sense, *T_x_*_0_ is the temperature at which crystallization is observed on the *B_s_*(*T*)–*T* curve to have a noticeable effect on the magnetic properties.

**Figure 6:**
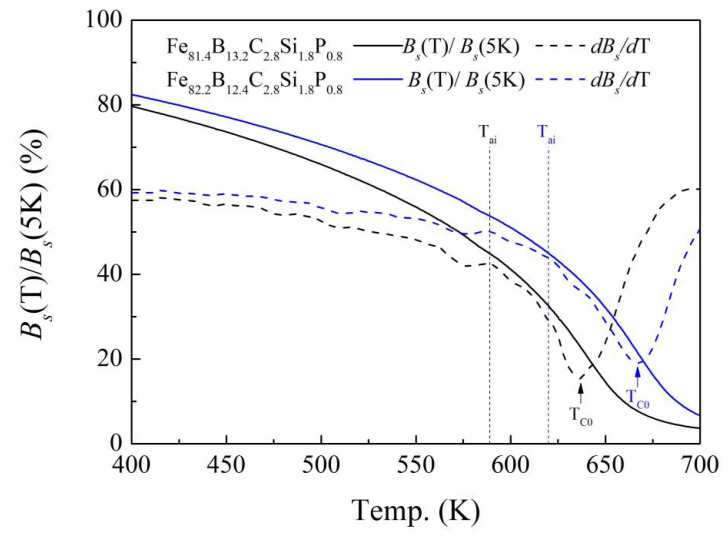
Reduced saturation flux density *B_s_*(*T*)/*B_s_*(5K) as a function of temperature for Fe_81.4_B_13.2_C_2.8_Si_1.8_P_0.8_ and Fe_82.2_B_12.4_C_2.8_Si_1.8_P_0.8_ amorphous alloys. Note that the dotted lines are the first derivative of *B_s_* with respect to temperature T for the alloys.

The shape of the *B_s_*(*T*)–*T* curve is related to the strength of the exchange interaction [18,24]. In order to visualize the declining rate of the *B_s_* with increasing temperature, the reduced saturation flux density *B_s_*(*T*)/*B_s_*(5K) as a function of temperature (*T*) and the derived first derivative of *B_s_* with respect to T are shown in Figure 6. For the Fe_81.4_ and Fe_82.2_ amorphous alloys, the *B_s_*(*T*)/*B_s_*(5K)–*T* and *dB_s_*/*dT*–*T* curves are similar. At first, the *B_s_*(*T*)/*B_s_*(5K) and *dB_s_*/*dT* decreased slowly with the increase in temperature. When the temperature continued rising to near the temperature denoted as T_ai_, at which *B_s_*(*T*)/*B_s_*(5K) = 45%, the value of *dB_s_*/*dT* began to decrease rapidly. Increasing the temperature further, *dB_s_*/*dT* decreased further and reached the lowest level. The corresponding temperature is denoted as *T_C_*_0_ (as shown in Figure 6). When the temperature was higher than *T_C_*_0_ but lower than *T_x_*_0_, *dB_s_*/*dT* gradually increased with the increase in temperature. Meanwhile, the decrease in *B_s_*(*T*)/*B_s_*(5K) developed slowly. In other words, *T_C_*_0_ represents the temperature at which the spontaneous magnetization of the alloy changed the most dramatically, i.e., the Curie temperature (the temperature at which ferromagnetism transforms into paramagnetism). The value of *T_C_*_0_ was indeed close to the Curie temperature obtained from the DSC curve (*T_C_*), as shown in Table 1.

Although the *B_s_*(*T*)–*T*, *B_s_*(*T*)/*B_s_*(5K)–*T*, and *dB_s_*/*dT*–*T* curves of the Fe_81.4_ and Fe_82.2_ amorphous alloys are similar, the *T_ai_* and *T_C_*_0_ of the Fe_82.2_ amorphous alloy were lower than those of the Fe_81.4_ amorphous alloy. This is due to the difference in the strength of the exchange interaction for the Fe_81.4_ and Fe_82.2_ amorphous alloys. The metalloid content of the Fe_82.2_ amorphous alloy was lower than that of the Fe_81.4_ amorphous alloy. The decrease in the metalloid content causes a reduction of the interatomic distance between the Fe atoms [18,24]. According to the Bethe–Slater curve [24], the strength of the exchange interaction will decrease with the decrease in the interatomic distances between Fe atoms. Therefore, the *dB_s_*/*dT* increases, resulting in a decrease in *T_ai_* and *T_C_*_0_.

By examining and analyzing the results shown in Figure 4, Figure 5 and Figure 6 , it is not difficult to find that *T_a_*_0_ is approximate to *T_ai_*. This may have been caused by the effect of spontaneous magnetization on the alloy relaxation process of these amorphous alloys. When the annealing temperature is lower than *T_ai_* but sufficient to allow short-distance movement of the atoms, the thermal motion of the atoms may cause changes in the local structure. On the one hand, long-term heat treatment at this temperature is enough to eliminate the influence of stress on *H_c_*, so that *H_c_* can be reduced compared to the cast state. On the other hand, the spontaneous magnetization is still high. Spontaneous magnetization will strengthen the local magnetic anisotropy [5,9,27], so it is difficult to achieve the optimal low coercivity after annealing. When the annealing temperature is higher than *T_ai_*, the decline in the d*B_s_*/d*T* value begins to accelerate, and the *B_s_*(*T*) decreases to less than half of the *B_s_* near 0 K (*B_s_*(*T*)/*B_s_*(5K) ≤ 45%), which demonstrates that the ferromagnetism of amorphous alloys is greatly weakened. The effect of the inner magnetic field generated by spontaneous magnetization on the relaxation behavior becomes negligible. The magnetic anisotropy of the alloy is reduced, and the soft magnetic properties are greatly improved. Based on the above analysis, we provide a simple way to determine the lowest temperature *T_ai_* (*B_s_*(*T_ai_*)/*B_s_*(5K) = 45%) of the optimum annealing temperature range for the Fe-based amorphous alloys with a relatively low Curie temperature, similar to the current amorphous alloys.

**Figure 7:**
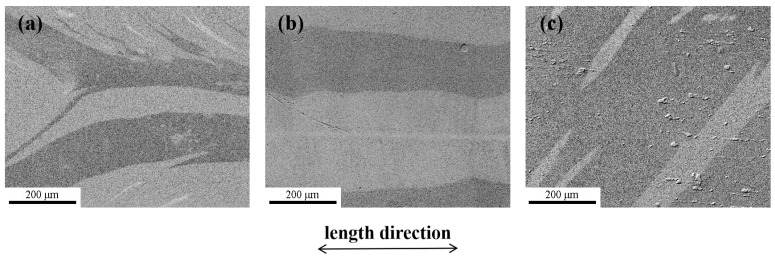
Magnetic domains in the demagnetized state for Fe_81.4_B_13.2_C_2.8_Si_1.8_P_0.8_ amorphous alloy samples: (**a**) as-spun, (**b**) NA-633 K, and (**c**) NA-603 K.

The magnetic domain structures of the amorphous alloy samples that experienced different annealing processes were characterized using a magneto-optical Kerr microscope, and the effect of spontaneous magnetization on the soft magnetic properties is discussed. Figure 7 shows the domain structures of the as-spun, NA-633 K-annealed and NA-603 K-annealed Fe_81.4_B_13.2_C_2.8_Si_1.8_P_0.8_ amorphous alloy samples in the demagnetized state. There were two types of typical domains in the as-spun Fe_81.4_ amorphous alloy sample (Figure 7a): wide-curved domains and narrow fingerprint domains. These are caused by tensile stress and compressive stress [5,28,29]. After normal annealing at 633 K, the domains of the sample appeared as a broad strip pattern oriented slightly away from the length direction of the amorphous ribbon, demonstrating a low domain energy, homogenous structure, and low stress state [30]. In contrast, more domain branches and rugged edges were present in the magnetic domain structure of the NA-603 K Fe_81.4_ amorphous alloy sample, and their direction deviated from the length direction, indicating a strong pinning effect. This phenomenon can be attributed to the magnetic anisotropy induced by spontaneous magnetization during the annealing process, because the annealing temperature was lower than *T_ai_* [5,9,27]. This shows that the annealing temperature above *T_ai_* is important for removing the pinning effect to obtain a low *H_c_*, as shown in Figure 4.

*T_am_* is the temperature related to the crystallization of amorphous alloys; above this, after annealing for a certain time, a volume fraction of α-Fe nanocrystalline would precipitate from the amorphous matrix of the alloys. Examples are shown in Figure 1: a few weak but sharp diffraction peaks corresponding to the α-Fe crystalline phase can be detected in the smooth-side XRD patterns of the Fe_81.4_ ribbons annealed at 663 K and the Fe_82.2_ ribbons annealed at 633 K for 90 min. The HRTEM image in Figure 2 shows that the as-spun Fe_82.2_ ribbon is completely amorphous with no pre-existing tiny α-Fe grains before annealing. Thus, the crystallization of Fe_82.2_ ribbons annealed at 633 K for 90 min is entirely due to annealing. It has been confirmed that partial crystallization at the surface increases the anisotropy, resulting in an increase in the *H_c_* and deterioration of the soft magnetic properties [31,32,33]. Additionally, the Curie temperature of the α-Fe cluster is higher than that of amorphous alloys, and it further increases with the cluster size and magnetic interaction [9,31], which aggravates the effect of spontaneous magnetization on the relaxation process.

It is well known that amorphous alloys are of a thermodynamic metastable state. They will eventually crystallize as long as the annealing time is long enough at a high enough temperature. Moreover, the higher the annealing temperature is, the shorter the time required for crystallization. Therefore, the *T_am_* is actually also related to the annealing time. When the annealing time is 90 min, crystallization of the Fe_82.2_ amorphous alloy occurs at 633 K, resulting in the *H_c_* increasing to 6.3 A/m. However, after annealing at 633 K for 60 min, the *H_c_* of the amorphous alloy is 1.4 A/m; that is, the highest temperature of the optimum annealing temperature range for 60 min is not lower than 633 K.

**Figure 8:**
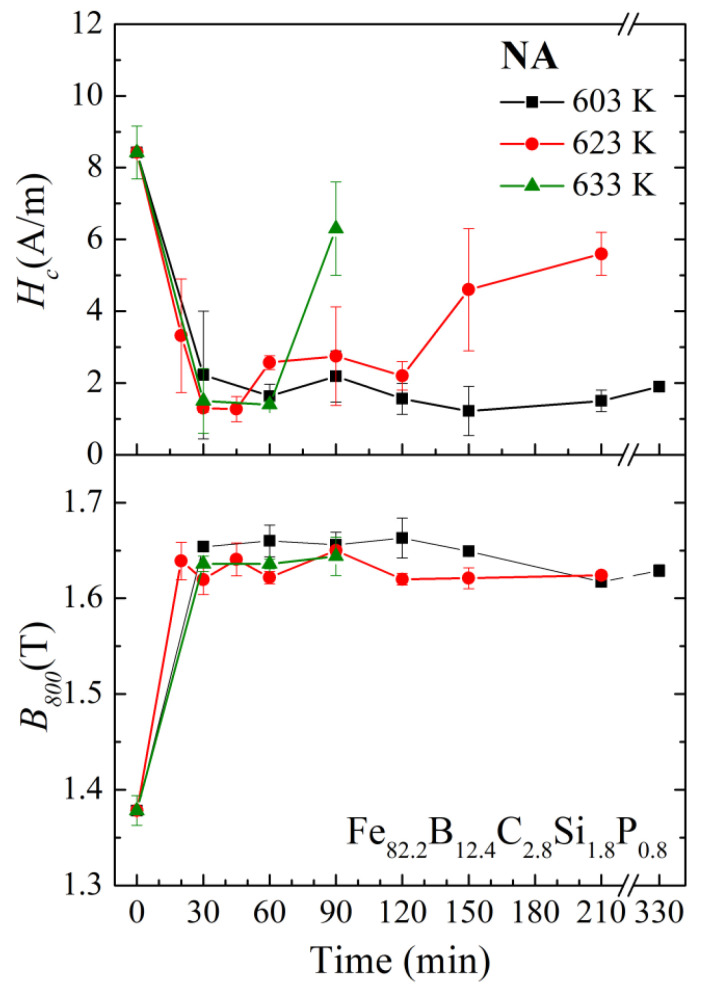
Annealing time dependence of *H_c_* and *B*_800_ for the Fe_82.2_B_12.4_C_2.8_Si_1.8_P_0.8_ amorphous alloy ribbons with normal annealing at different temperatures.

Figure 8 illustrates the annealing time dependence of *H_c_* and *B*_800_ for the Fe_82.2_B_12.4_C_2.8_Si_1.8_P_0.8_ amorphous alloy ribbons with normal annealing at different temperatures. Three different annealing temperatures were selected for the Fe_82.2_ amorphous alloy: *T_a_*_1_ = 603 K, *T_a_*_2_ = *T_am_* (90 min) = 623 K, and *T_a_*_3_ = 633 K, respectively. Overall, with the increase in the annealing time, the *B_800_* of the alloys increased initially and then remained relatively stable. When *T_a_*_3_ = 633 K, the *H_c_* first decreased rapidly as the annealing time increased, then remained stable for a short time (30–60 min), and rose quickly after the annealing time reached 90 min. When *T_a_*_2_ = 623 K, the *H_c_* showed a concave shape with respect to annealing time: the *H_c_* first decreased rapidly, then decreased to 1.3 A/m after 30 min, and remained below 3 A/m within 30–120 min. Up to an annealing time of 150 min, the *H_c_* rose to 4.6 A/m. However, when the annealing temperature decreased to *T_a_*_1_ (603 K), the coercivity *H_c_* of the annealed samples exhibited very strong stability with the increase in annealing time. Even when the annealing time was extended to 330 min, the *H_c_* was still only 1.9 A/m. For the Fe_82.2_ amorphous alloy, *T_a_*_1_ was slightly higher than *T_ai_* (*T_a_*_1_ − *T_ai_* < 15 K).

**Figure 9:**
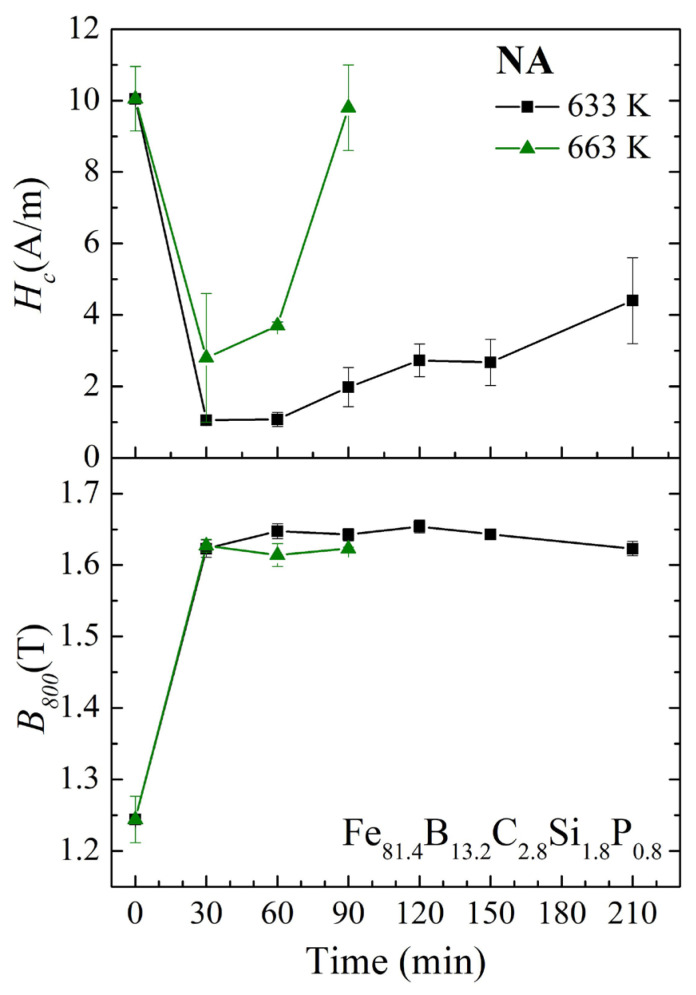
Annealing time dependence of *H_c_* and *B*_800_ for the Fe_81.4_B_13.2_C_2.8_Si_1.8_P_0.8_ amorphous alloy ribbons with normal annealing at different temperatures.

The annealing time dependence of *H_c_* and *B_800_* for the Fe_81.4_B_13.2_C_2.8_Si_1.8_P_0.8_ amorphous alloy ribbons with normal annealing at different temperatures is shown in Figure 9. Two different annealing temperatures were selected for the Fe_81.4_ amorphous alloy, which were *T_a_*_4_ = 633 K and *T_a_*_5_ = 663 K, respectively. For both annealing temperatures, with the increase in annealing time, the *B*_800_ increased rapidly at first and then became stable. When the annealing temperature was *T_a_*_4_ = 633 K, the *H_c_* rapidly decreased to about 1 A/m and then slowly increased. The *H_c_* remained at 2.7 A/m after 150 min of annealing. However, when the annealing temperature increased to *T_a_*_5_ = 663 K, the *H_c_* remained relatively stable only within 30–60 min, and at this time, the *H_c_* was 2.8–3.7 A/m, which was significantly higher than that at 633 K. Although the *H_c_* of the Fe_81.4_ amorphous alloy shown in Figure 9 is higher than that of the Fe_82.2_ amorphous alloy shown in Figure 8, it can also be seen that when the annealing temperature is selected properly (slightly higher than *T_ai_*), the soft magnetic properties can remain relatively stable for a longer period of time.

**Figure 10:**
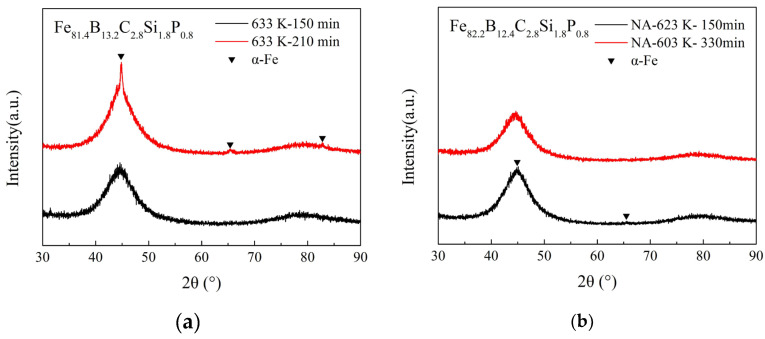
Smooth-side XRD patterns of ribbons with different annealing times and temperatures for (**a**) Fe_81.4_B_13.2_C_2.8_Si_1.8_P_0.8_ and (**b**) Fe_82.2_B_12.4_C_2.8_Si_1.8_P_0.8_ amorphous alloys.

The increase in *H_c_* in the long-term annealed samples shown in Figure 8 and Figure 9 is also due to the formation of nanocrystalline clusters and the growth of these clusters. Figure 10 shows the smooth-side XRD patterns of the ribbons with different annealing times and temperatures for the Fe_81.4_ and Fe_82.2_ amorphous alloys. As shown in Figure 10, few weak diffraction peaks corresponding to the α-Fe crystalline phase can be detected in the smooth-side XRD patterns of the Fe_81.4_ ribbons annealed at 633 K for 210 min and the Fe_82.2_ ribbons annealed at 623 K for 150 min.

**Figure 11:**
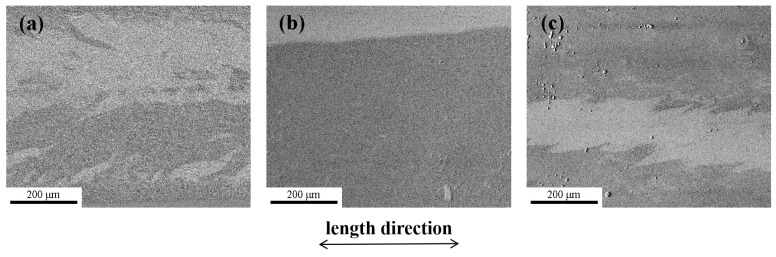
Magnetic domains in the demagnetized state for the Fe_82.2_B_12.4_C_2.8_Si_1.8_P_0.8_ amorphous alloy samples: (**a**) as-spun, (**b**) NA-623K-45 min, and (**c**) NA-623K-210 min.

Figure 11 shows the magnetic domain structures of the as-spun, NA-623K-45 min-annealed and NA-623K-210 min annealed Fe_82.2_B_12.4_C_2.8_Si_1.8_P_0.8_ amorphous alloy samples in the demagnetized state. Wide-curved domains and narrow fingerprint domains, caused by tensile stress and compressive stress [5,29], are observed in the as-spun Fe_82.2_ amorphous alloy sample (Figure 11a). The domains of the sample annealing at 623 K for 45 min appear as a broad strip pattern oriented slightly away from the length direction of the amorphous ribbon, demonstrating the low domain energy, homogenous structure, and low stress state. However, for the sample annealed at 623K for 210 min, the magnetic domain structure with rugged edges can be clearly observed (Figure 11c), which is quite different from those in the NA-623 K-45 min annealed sample (Figure 11b). This indicates that a strong pinning effect exists, induced by partial crystallization resulting from long-term annealing (see Figure 10b) [5,9,27,32].

The above results indicate that when the annealing temperature is selected properly (slightly higher than *T_ai_*), excellent and stable soft magnetic properties of amorphous alloys can be obtained through annealing even if the annealing is maintained for a very long time. Annealing slightly above *T_ai_* could eliminate the influence of stress and the internal magnetic field on the *H_c_*. In addition, a relatively low annealing temperature could reduce the possibility of crystallization. This result is of great significance for designing an annealing process for industrial applications.

## 4. Conclusions

The effects of annealing process parameters, including the annealing temperature and time, on the soft magnetic properties and magnetic domain structures of Fe_81.4_B_13.2_C_2.8_Si_1.8_P_0.8_ and Fe_82.2_B_12.4_C_2.8_Si_1.8_P_0.8_ amorphous alloys were systematically investigated, and the conclusions are summarized as follows:

(1) Fe_81.4_B_13.2_C_2.8_Si_1.8_P_0.8_ and Fe_82.2_B_12.4_C_2.8_Si_1.8_P_0.8_ amorphous alloys were designed and prepared. This revealed that a lowest and highest temperature, denoted as *T_a_*_0_ and *T_am_*, respectively, exists for the optimum annealing of the amorphous alloys. The *H_c_* of the Fe_81.4_ and Fe_82.2_ amorphous alloys annealed at *T_a_*_0_–*T_am_* for 90 min was 1.3–2.7 A/m, together with the *B*_800_ of 1.64–1.66 T. *T_a_*_0_ is determined by the variation in the magnetic properties with temperature. *T_am_* is the temperature related to the crystallization of amorphous alloys.
(2) It was found that on the *B_s_*(*T*)–*T* curve, there is a temperature *T_ai_* at which the *dB_s_*/*dT* begins to decrease rapidly, and *B_s_*(*T_ai_*)/*B_s_*(5K) = 45%. When the amorphous alloys were annealed slightly above *T_ai_*, the effect of the inner magnetic field generated by spontaneous magnetization on the relaxation behavior became very weak. That is, the temperature *T_ai_* could be employed as a characteristic temperature. Slightly above *T_ai_*, an optimized annealing temperature *T_a_*_0_ for the Fe-based amorphous alloys with a relatively low Curie temperature, similar to the studied alloys, could be determined quickly.
(3) When the annealing temperature was selected properly (slightly higher than *T_ai_*), the soft magnetic properties of amorphous alloys could remain excellent and stable even if annealed for a very long time. The *H_c_* of the Fe_82.2_B_12.4_C_2.8_Si_1.8_P_0.8_ amorphous alloy annealed at *T_a_*_1_ = 603 K was only 1.9 A/m, while the annealing time was extended to 330 min.

## Figures and Tables

**Table 1:** Several temperature parameters mentioned in this article for Fe_81.4_B_13.2_C_2.8_Si_1.8_P_0.8_ and Fe_82.2_B_12.4_C_2.8_Si_1.8_P_0.8_ amorphous alloys.

Alloy	*T_a_*_0_ (K)	*T_am_* (K)	*T_ai_* (K)	*T_C_*_0_ (K)	*T_x_*_0_ (K)	*T_C_* (K)	*T_x_* (K)
Fe_81.4_B_13.2_C_2.8_Si_1.8_P_0.8_	623	653	620	667	744	661	776
Fe_82.2_B_12.4_C_2.8_Si_1.8_P_0.8_	593	623	589	637	709	635	766

*T_a_*_0_ and *T_am_* are the lowest temperature and the highest temperature of the optimum annealing temperature range, as shown in Figure 4; *T_x_*_0_, *T_ai_*, and *T_C_*_0_ are the characteristic temperatures, as shown in Figure 5 and Figure 6; *T_C_* and *T_x_* are the Curie temperature and the onset temperature of crystallization, determined from the DSC curves shown in Figure 3.

## Data Availability

The raw data supporting the conclusions of this article will be made available by the authors upon request.
